# Cholinesterase Enzymes Inhibitors from the Leaves of *Rauvolfia Reflexa* and Their Molecular Docking Study

**DOI:** 10.3390/molecules18043779

**Published:** 2013-03-25

**Authors:** Mehran Fadaeinasab, A. Hamid A. Hadi, Yalda Kia, Alireza Basiri, Vikneswaran Murugaiyah

**Affiliations:** 1 Department of Chemistry, Faculty of Science, University of Malaya, 50603 Kuala Lumpur, Malaysia; E-Mail: mehranfadaie_n@yahoo.com; 2 School of Chemical Sciences, Universiti Sains Malaysia, 11800 Penang, Malaysia; E-Mail: Kia.yalda@yahoo.com; 3 School of Pharmaceutical Sciences, Universiti Sains Malaysia, 11800 Penang, Malaysia; E-Mails: diba115920@gmail.com (A.B.); vicky@usm.my (V.M.)

**Keywords:** Apocynaceae, *Rauvolfia reflexa*, acetylcholinesterase, butyrylcholinesterase, molecular docking

## Abstract

Plants of the Apocynaceae family have been traditionally used in the treatment of age-related brain disorders. *Rauvolfia reflexa*, a member of the family, has been used as an antidote for poisons and to treat malaria. The dichloromethane, ethanol and methanol extracts from the leaves of *Rauvolfia reflexa* showed potential acetylcholinesterase (AChE) and butyrylcholinesterase (BChE) inhibitory activities, with IC_50_ values in the 8.49 to 52.23 g/mL range. Further cholinesterase inhibitory-guided isolation of these extracts afforded four bioactive compounds, namely: (*E*)-3-(3,4,5-trimethoxyphenyl)acrylic acid (**1**), (*E*)-methyl 3-(4-hydroxy-3,5-dimethoxyphenyl) acrylate (**2**), 17-methoxycarbonyl-14-heptadecaenyl-4-hydroxy-3-methoxycinnamate (**3**) and 1,2,3,4-tetrahydro-1-oxo-β-carboline (**4**). The isolated compounds showed moderate cholinesterase inhibitory activity compared to the reference standard, physostigmine. Compounds **1** and **2** showed the highest inhibitory activity against AChE (IC_50_ = 60.17 µM) and BChE (IC_50_ = 61.72 µM), respectively. Despite having similar molecular weight, compounds **1** and **2** were structurally different according to their chemical substitution patterns, leading to their different enzyme inhibition selectivity. Compound **2** was more selective against BChE, whereas compound **1** was a selective inhibitor of AChE. Molecular docking revealed that both compounds **1** and **2** were inserted, but not deeply into the active site of the cholinesterase enzymes.

## 1. Introduction

Alzheimer’s disease (AD) is the leading cause of dementia among older people [1]. It is a chronic and progressive neurodegenerative disease characterized neuropathologically by the extracellular deposition of β-amyloid aggregates and intraneuronal neurofibrillary tangles. Neuronal loss at the affected regions causes deficit in production of the neurotransmitter acetylcholine, leading to cortical cholinergic dysfunction [2].

Based on the cholinergic hypothesis, acetylcholinesterase inhibitors were developed to sustain or enhance the acetylcholine levels. The crucial role of cholinesterases in neural transmission makes them a primary target of a large number of cholinesterase-inhibiting drugs and toxins [3], which toxins are useful for agricultural purposes and novel drugs that need to be prepared, although there is less interest in the new toxins [4]. Acetylcholinesterase inhibitors such as donepezil, rivastigmine and galantamine are currently still the best available pharmacotherapy for AD patients [5]. Natural products have contributed greatly as sources in drug discovery for Alzheimer’s disease. For examples, excellent acetylcholinesterase inhibitors such as physostigmine and galantamine have been isolated from *Physostigma venenosum* and *Galanthus nivalis*, respectively. Therefore, the search for new cholinesterase inhibitors from natural products is of great interest and ongoing in many parts of the World.

Plants of the Apocynaceae family such as *Tabernaemontana heterophylla* Vahl were traditionally used in age related brain disorders by the Tukano Indians, in which a tea of the leaves is given for the old folks who are slow and forgetful. Similarly, *Parahamcornia amapa* was used against general debility in the Brazilian Amazon [6].

*Rauvolfia reflexa* is a member of the Apocynaceae family. Traditionally, it is believed to have strong antiplasmodial activity. In Tanzania, a decoction of the leaves is used to treat malaria [7]. To date, there are very few publications on this plant, mostly related to the isolation and characterization of its chemical constituents. In the present study, extracts and chemical constituents of *Rauvolfia reflexa* were evaluated for their potential cholinesterase inhibitory activity. Subsequently, a molecular docking study was undertaken to investigate the molecular interactions between the inhibitory compounds and enzymes.

## 2. Results and Discussion

### 2.1. Bioactivity-Guided Isolation

Among the three extracts, the dichloromethane and ethanol ones exhibited good inhibitory activity against both enzymes, while the methanol extract showed moderate inhibitory activity against acetylcholinesterase. Thus, all three extracts were selected for further purification in the search for bioactive chemical constituents. 

**Figure 1:**
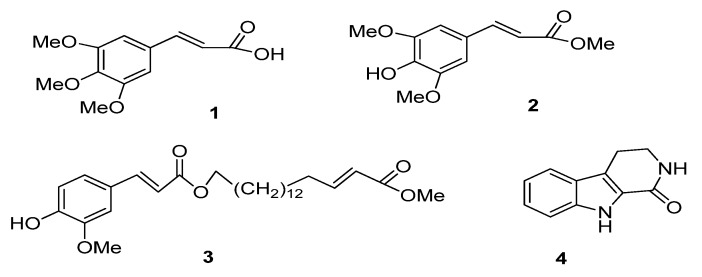
Compounds isolated from leaves of *R. reflexa*.

**Figure 2:**
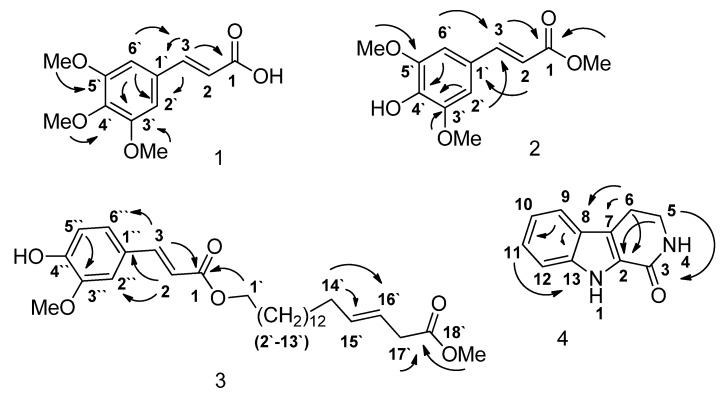
HMBC correlations of compounds **1** to **4**.

The dichloromethane extract that showed the highest enzyme inhibitory activity against both AChE and BChE afforded compounds **1** (13 mg, 0.00065%, Figure 1) and **2** (19 mg, 0.00095%, Figure 1). Compounds **3** (17 mg, 0.00085%, Figure 1) and **4** (12 mg, 0.0006%, Figure 1) were isolated from the ethanolic and methanolic extracts, respectively (Figure 2). The structures of **1**–**4** were confirmed by comparison of their NMR, MS, UV and IR spectra with those reported previously [8,9,10,11]. The HMBC correlations of compounds **1**–**4** are given in Figure 2.

### 2.2. Cholinesterase Enzymes Inhibitory Activity

Initial cholinesterase enzymes inhibitory activity of the extracts was evaluated at 50 μg/mL while for the isolated chemical constituents this was done at 10 μg/mL. Among the extracts, the dichloromethane extract had the highest enzyme inhibitory activity against both AChE and BChE, with inhibition values of 80.79% and 90.11%, respectively. The ethanol extract also showed good inhibitory activity of more than 50% against both enzymes, but methanol extract only showed more than 50% inhibition against AChE.

Among the isolated chemical constituents, **2** had the highest enzyme inhibitory activity against BChE, with an inhibition value of 91.29%, while the other three compounds showed inhibitions of less than 50% against BChE. On the other hand, for AChE, **2**–**4** had similar inhibition values of 40.36%, 41.05% and 41.27%, respectively.

Table 1 summarizes the IC_50_ and selectivity index of the *R. reflexa* extracts. The IC_50_ values obtained were in agreement with the initial screening data. Dichloromethane crude extract was the most active extract, with IC_50_ values of 14.65 μg/mL and 8.49 μg/mL against AChE and BChE, respectively. In the present study, dichloromethane and ethanolic extract of *R. reflexa* showed good enzyme inhibitory activity against both AChE and BChE, while methanolic extract had moderate activity against AChE. Both dichloromethane and ethanolic extracts were more selective towards BChE inhibition than AChE, in contrast to the methanol crude extract.

**Table 1:** IC_50_ values of *Rauvolfia reflexa* extracts for inhibitory activities on cholinesterase enzymes.

Extract	AChE inhibition, IC_50_ (μg/mL)	BChE inhibition, IC_50_ (μg/mL)	Selectivity for	
			AChE ^a^	BChE ^b^
Dichloromethane	14.65 ± 0.32	8.49 ± 0.92	0.58	1.73
Ethanol	38.40 ± 0.15	26.47 ± 2.05	0.79	1.26
Methanol	52.23 ± 4.02	ND	-	-

Data presented as Mean ± SD (n = 3); ^a^ Selectivity for AChE is defined as IC_50_(BChE)/IC_50_(AChE); ^b^ Selectivity for BChE is defined as IC_50_(AChE)/IC_50_(BChE).

The IC_50_ values and selectivity index of the isolated chemical constituents and reference inhibitor, physostigmine are shown in Table 2. For BChE, the IC_50_ was only determined for **2** as the others had less than 50% inhibition at 10 μg/mL. However, its IC_50_ value was higher than the dichloromethane extract, indicating that the inhibitory activity of dichloromethane could be potentiated by other compounds present in the dichloromethane extract. The IC_50_ value of **2** against BChE was 61.72 μM, which was approximately 105 times less potent than physostigmine.

**Table 2:** IC_50_ values of *Rauvolfia reflexa* chemical constituents for inhibitory activities on cholinesterase enzymes.

Compound	AChE inhibition, IC50		BChE inhibition, IC50		Selectivity for	
	μg/mL	μM	μg/mL	μM	AChE a	BChE b
**1**	14.32 ± 0.82	60.17 ± 14.45	ND	-	-	-
**2**	37.63 ± 1.42	158.06 ± 5.9	14.69 ± 1.22	61.72 ± 5.14	0.39	2.56
**3**	48.99 ± 2.86	97.37 ± 5.64	ND	-	-	-
**4**	15.52 ± 0.68	83.38 ± 3.67	ND	-	-	-
Physostigmine	0.046	0.17	0.162	0.59	3.47	0.29

Data presented as Mean ± SD (n = 3); ^a^ Selectivity for AChE is defined as IC_50_(BChE)/IC_50_(AChE); ^b^ Selectivity for BChE is defined as IC_50_(AChE)/IC_50_(BChE).

For AChE inhibition at 10 μg/mL concentration, compounds **2**–**4** showed almost similar inhibitory activity, while compound **1** had a much lower inhibition. However, comparison of their IC_50_ values showed that compound **1** had the lowest IC_50_ among all the compounds. Its activity was moderate and on molar basis, it is approximately 354 times less potent than physostigmine. The inhibitory activities of compounds **1** and **4** against AChE were more potent than their respective extracts, dichloromethane and methanolic extracts, whereas the inhibitory activity of compound **3** was less potent than that of ethanolic extract.

In general, compared to physostigmine, the four isolated compounds showed moderate AChE inhibitory activity, while only **2** had good inhibitory activity against BChE. Despite their moderate activity, these compounds could serve as leads for synthesis of potential analogues with improved inhibitory activity. An interesting observation in the present study is the inhibitory activity between compounds **1** and **2**. Both of them had the same molecular weight but structurally different by their chemical substitution. Compound **1** is an acid whereas compound **2** is an ester. Compound **2** was found to be much more active than compound **1** against BChE. In addition, the relative selectivity of compound **2** is more on BChE than AChE, whereas for compound **1**, this is reversed. 

### 2.3. Molecular Docking of Bioactive Compounds **1** and **2**

**Figure 3:**
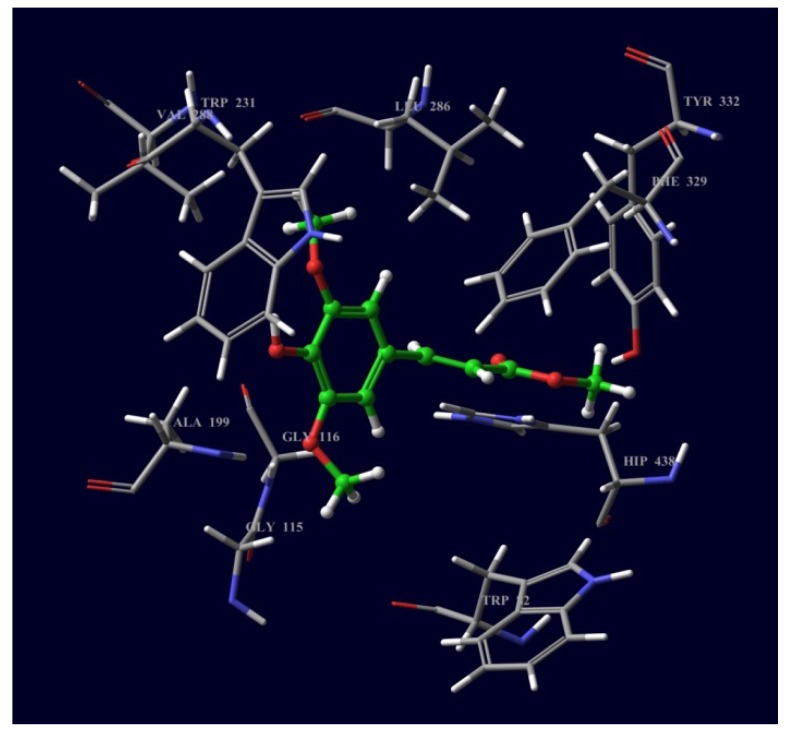
Compound **1** docked into the active site of *Tc*AChE.

**Figure 4:**
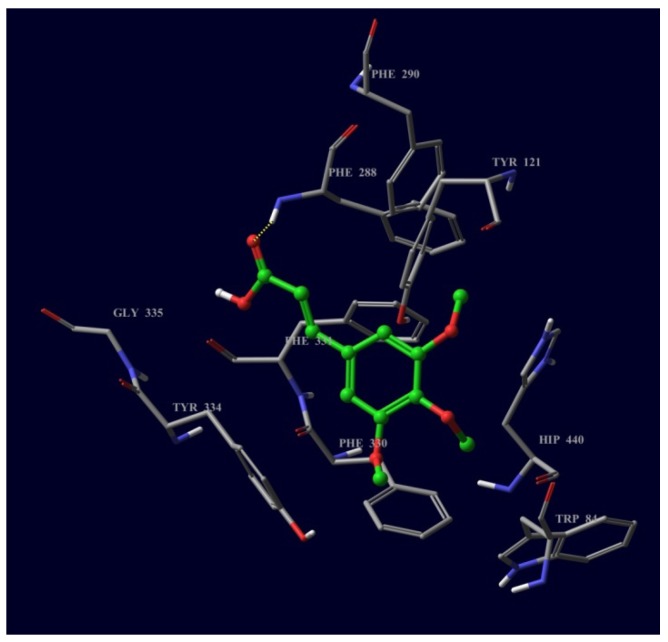
Compound **2** docked into active site of BChE.

Figure 3 and Figure 4 show compounds **1** and **2** docked onto the *Tc*AChE and BChE enzymes, respectively. For compound **1**, the major bindings were π-π stacking, hydrogen bonding, and hydrophobic interactions. The aromatic ring of 1 strongly stacked against the aromatic moieties of side chain residues such as Tyr 334 and Phe 331. A hydrogen bond (2.1 Å) between the N-H moiety of the Phe 288 residue and the C=O moiety of compound **1** anchors the ligand to the acyl binding pocket site of the active site gorge. This ligand also shows hydrophobic interactions with Trp 84, Phe 330 at the choline binding site, Tyr 121 at a peripheral binding site and Phe 290 at the choline binding site. 

When compound **2** is docked onto the BChE receptor, there were indications of hydrogen bonds, hydrophobic, mild polar interactions and π-π stacking. His 438 at the catalytic triad showed a strong π-π stacking interaction with the aromatic moiety of **2**. Phe 329 at the choline binding site and Trp 231, Val 288 and Leu 286 at the peripheral anionic site showed hydrophobic interactions mainly with the methoxy moieties of the ligand. Ala 199 and Gly 116 at the oxyanion hole showed strong hydrogen bonds (2.28 and 2.02 Å) with the methoxy moiety of the ligand. These results indicate that the compounds **1** and **2** were inserted, but not deeply, into the active site cavity, which is commonly observed in drugs used for treatment of AD [12]. 

## 3. Experimental

### 3.1. Plant Materials

The plant materials (leaves) of *Rauvolfia reflexa* were collected from the Pergau Hydroelectric Station, Jeli, Kelantan, Malaysia in July 2009. The botanical identification was made by Mr. Teo Leong Eng, Faculty of Science, University of Malaya. A voucher specimen (KL 4900) was deposited at the Herbarium of Chemistry Department, Faculty of Science, University of Malaya.

### 3.2. Enzymes and Chemicals

Acetylthiocholine iodide (ATCl), acetylcholinesterase from electric eel (AChE), 5,5'-dithiobis[2-nitrobenzoic acid] (DTNB), butyrylcholinesterase from equine serum (BChE), *S*-butyrylthiocholine chloride and physostigmine were purchased from Sigma Chemicals (St. Louis, MO, USA). All the other reagents used were of analytical grade.

### 3.3. General Experimental Procedures

TLC was performed on aluminum and glass plates pre-coated with silica gel 60 F_254_ (Merck, Darmstadt, Germany). ^1^H-NMR and ^13^C-NMR spectra were determined in CDCl_3_ on a JEOL JNM-FX400 spectrometer (Tokyo, Japan), UV spectra were recorded on a Shimadzu UV-160A spectrophotometer (Kyoto, Japan) using MeOH as solvent. MS was obtained on an Agilent 6530 mass spectrometer (Santa Clara, CA, USA). The IR spectrum was measured on a Perkin-Elmer RX 1 spectrometer (Waltham, MA, USA) for the 4,000–400 cm^−1^ frequencies range. 

### 3.4. Preparation of Extract

The dried leaves of *R*. *reflexa* (2 kg) were first defatted in *n*-hexane (2,000 mL) for 48 h. Then, the extract was filtered and dried on the rotary evaporator. The residue was sequentially re-extracted with dichloromethane (CH_2_Cl_2_, 2,000 mL, two times), ethanol (EtOH, 2,000 mL, two times) and methanol (MeOH, 2,000 mL, two times) and the resulting filtrates were dried under reduced pressure by a rotary evaporator at 40 °C to yield CH_2_Cl_2_ (15 g, 0.75% yield), EtOH (10 g, 0.50% yield) and MeOH (14 g, 0.70% yield) extracts, respectively.

### 3.5. Isolation and Characterization of Bioactive Compounds

Dichloromethane extract (15 g) was chromatographed on a silica gel 60 column (40–63 μm particle size) and eluted sequentially with CH_2_Cl_2_/*n*-hexane/MeOH mixtures (80:10:10 → 0:0:100). Eluates were collected and those displaying similar R_f_ values on TLC were pooled to yield 18 fractions. Fraction 12 was subjected to a preparative silica gel TLC with a solvent system of CH_2_Cl_2_/MeOH (90:10) to yield **1** (13 mg, 0.00065%) and (**2**, 19 mg, 0.00095%).

Ethanolic extract (10 g) was chromatographed on a silica gel 60 column (40–63 μm particle size) and was eluted sequentially with CH_2_Cl_2_/*n*-hexane/EtOH mixtures (85:10:5 → 0:0:100). Eluates were collected and those displaying similar R_f_ values on TLC were pooled to yield 14 fractions. Fraction 9 was subjected to a preparative silica gel TLC with a solvent system of CH_2_Cl_2_/EtOH (90:10) to yield **3** (17 mg, 0.00085%).

MeOH (14 g) extract was chromatographed on a LH-20 Sephadex column and was eluted sequentially with CHCL_3_/MeOH mixtures (80:20 → 0:100). Eluates were collected and those displaying similar R_f_ values on TLC were pooled to yield 22 fractions. Fraction 7 was further subjected to a preparative TLC on a reversed-phase silica gel with a solvent system of water/MeOH (30:70) to yield **4** (12 mg, 0.0006%).

### 3.6. Characterization Data

*(E)-3-(3,4,5-Trimethoxyphenyl) acrylic a*cid (**1**): white λνν UV (MeOH) *λ*_max_ 225, 307 nm; IR (CHCl_3_) *ν*_max_ 3338, 1731 cm^−1^; ESIMS *m*/*z* 238.09 (M)^+^(100), [calcd. for C_12_H_14_O_5_]; ^1^H-NMR (CDCl_3_) *δ* 3.87 s, 3H, OCH_3_-3'), 3.87 (s, 3H, OCH_3_-4'), 3.87 (s, 3H, OCH_3_-5'), 5.50 (bs, 1H, OH-1), 6.38 (d, *J* = 15.6 Hz, 1H, H-2), 6.74 (s, 1H, H-6'), 6.74 (s, 1H, H-2'), 7.58 (d, *J* = 15.6 Hz, 1H, H-3); ^13^C-NMR (CDCl_3_): 53.4, 56.1, 60.4, 105.1, 106.5,118.6 122.7, 130.2, 142.6, 153.4, 153.4, 167.5.

*(E)-methyl 3-(4-hydroxy-3,5-dimethoxyphenyl) acrylate* (**2**): white amorphous solid; UV (MeOH) *λ*_max_ 196, 273 and 328 nm; IR (CHCl_3_) *ν*_max_ 3401, 1704 cm^−1^; ESIMS *m*/*z* 238.09 (M)^+^(100), [calcd. for C_12_H_14_O_5_]; ^1^H-NMR (CDCl_3_) *δ* 3.79 (s, 3H, OCH_3_-1), 3.91 (s, 3H, OCH_3_-5'), 3.92 (s, 3H, OCH_3_-3'), 5.80 (bs, 1H, OH-4'), 6.29 (d, *J* = 16.0 Hz, 1H, H-2), 6.77 (s, 1H, H-6'), 6.77 (s, 1H, H-2'), 7.59 (d, *J* = 16.0 Hz, 1H, H-3); ^13^C-NMR (CDCl_3_): 51.7, 56.4, 56.4, 105.1, 105.1, 115.6, 125.9, 137.1, 145.2, 147.2, 167.6.

*17-Methoxycarbonyl-14-heptadecaenyl-4-hydroxy-3-methoxy cinnamate* (**3**): white amorphous solid; UV (MeOH) *λ*_max_ 196, 273 and 328 nm; IR (CHCl_3_) *ν*_max_ 3431, 1711 and 1738 cm^−1^; ESIMS *m*/*z* 488.3 (M)^+^(100), [calcd. for C_29_H_44_O_6_]; ^1^H NMR (400 MHz, CDCL_3_) *δ* 1.28–1.58 (s, 13H, H-2'-13'), 2.10 (d, *J* = 5.4 Hz 2H, H-14'), 2.31 (m, 2H, H-17'), 3.64 (s, 3H, OCH_3_-18'), 4.10 (t, *J* = 6.8 Hz, 2H, H-1'), 5.32 (m, 1H, H-15'), 5.32 (m, 1H, H-16'), 5.90 (s,3H, OCH_3_-3''), 5.91 (bs, OH-4''), 6.27 (d, *J* = 16 Hz, 1H, H-2), 6.90 (d, *J* = 8.2 Hz, 1H, H-5''), 7.01 (s, 1H, H-2''), 7.10 (d, *J* = 8.2 Hz, 1H, H-6''), 7.50 (d, *J* = 16 Hz, H-3); ^13^C-NMR (100 MHz, CDCL_3_): 29.2–29.7, 29.8, 34.0, 51.5, 56.0, 64.6, 114.7, 109.3, 115.7, 123.1, 127.1, 129.9, 130.1, 144.7, 146.8, 147.9, 167.4.

*1,2,3,4-Tetrahydro-1-oxo-β-carboline* (**4**): brownish amorphous solid; ${\left[\alpha \right]}_{D}^{24}$: −16 (*c* 0.05, CHCl_3_); UV (MeOH) *λ*_max_ 246, 302 nm; IR (CHCl_3_) *ν*_max_ 3435, 1730 cm^−1^; ESIMS *m*/*z* 186.08 (M)^+^(100) [calcd. for C_11_H_10_N_2_O]; ^1^H-NMR (CDCl_3_) *δ* 3.06 (t, *J* = 6.8, 14.2 Hz, 2H, H-6), 3.71 (dt, *J* = 2.7, 7.3 Hz, 2H, H-5), 5.76 (s, 1H, NH-4), 7.15 (dt, *J* = 0.9, 7.2 Hz, 1H, H-10), 7.31 (dt, *J* = 0.8, 6.9 Hz, 1H, H-11), 7.45 (d, *J* = 8.2 Hz 1H, H-9), 7.58 (d, *J* = 8.2 Hz, 1H, H-12), 9.26 (s, 1H, NH-1); ^13^C-NMR (CDCl_3_): 20.9, 42.31, 112.5, 120.0, 120.4, 120.4, 125.3, 126.2, 126.2, 137.2, 163.1.

### 3.7. Cholinesterase Enzymes Inhibitory Assay

Cholinesterase enzymes inhibitory potential of test samples was evaluated using Ellman’s microplate assay following the method described by Ahmed and Gilani [13] with slight modifications. Physostigmine was used as positive control. Test samples and physostigmine were prepared in dimethyl sulfoxide (DMSO). The concentration of DMSO in final reaction mixture was 1%. At this concentration, DMSO has no inhibitory effect on both acetylcholinesterase and butyrylcholinesterase enzymes [14].

For acetylcholinesterase (AChE) inhibitory assay, 140 μL of 0.1 M sodium phosphate buffer (pH 8) was first added to a 96-wells microplate followed by 20 μL of test samples and 20 μL of 0.09 units/mL acetylcholinesterase enzyme. After 15 min of pre-incubation at 25 °C, 10 μL of 10 mM 5,5'-dithiobis (2-nitrobenzoic acid) was added into each well, followed by 10 μL of 14 mM acetylthiocholine iodide. After the initiation of enzymatic reaction, absorbance of the coloured end-product was measured for 15 min using Tecan Infinite 200 Pro Microplate Spectrophotometer (Männedorf, Switzerland) at 417 nm.

For butyrylcholinesterase (BChE) inhibitory assay, the same procedures as described above were followed except for the use of enzyme and substrate, which were BChE from equine serum and *S*-butyrylthiocholine chloride, respectively. Each test was conducted in triplicate. Absorbencies of the test samples were corrected by subtracting the absorbance of their respective blank. A set of five concentrations was used to estimate the 50% inhibitory concentration (IC_50_). Percentage inhibition was calculated using the following formula:

$$\text{Percentage inhibition (\%) }=\;\frac{\text{Absorbance of control }-\text{ Absorbance of test sample}}{\text{Absorbance of control}}\times 100\%$$

### 3.8. Molecular Docking

Among the isolated compounds, compounds **1**, **2** had the highest inhibitory activities on AChE and BChE, respectively. Thus, both compounds were selected for further molecular docking evaluation to explore possible interactions between these compounds and the active sites of the enzymes. Molecular docking study was performed using Glide (version 5.7, Schrödinger, LLC, New York, NY, USA, 2011). 

Compounds **1** and **2** were docked onto the active site of *Tc*AChE derived from three-dimensional structure of the enzyme complex with anti-Alzheimer’s drug, E2020 (Aricept™) (PDB ID: 1EVE) and to BChE derived from complex of the enzyme with Tabun analogue (PDB code: 2WIJ). Water molecules and hetero groups were deleted from receptor beyond the radius of 5 Å of reference ligand (E202 or Tabun), resulting protein structure refined and minimized by Protein Preparation Wizard using OPLS-2005 force field. The Receptor Grid Generation program were used to prepare AChE and BChE grids and all the ligands were optimized by the LigPrep program by using the OPLS-2005 force field to generate the lowest energy states of ligands.

## 4. Conclusions

In the present study, cholinesterase enzyme inhibitory activities of extracts and bioactive chemical constituents of *Rauvolfia reflexa* were investigated. Among the extracts, the dichloromethane and ethanol ones showed potential inhibitory activity against both AChE and BChE. The isolated chemical compounds showed moderate to weak inhibitory activity against both enzymes, compared to that of physostigmine, with compounds **1** and **2** having the most potent activity against AChE and BChE, respectively. These compounds were found to have contrasting cholinesterase enzymes inhibitory activities despite their similar molecular weight (differing in their chemical substitution patterns). Compound **2** showed selective inhibitory activity against BChE, whereas compound **1** was a more selective inhibitor of AChE. Both bioactive compounds were inserted into but did not fill the active site of the cholinesterase enzymes.

## References

[1] Racchi M., Mazzucchelli M.E., Porello G.L., Govoni S. (2004). Acetylcholinesterase inhibitors: Novel activities of old molecules. Pharmacol. Res..

[2] Massoud G., Gauthier S. (2010). Update on the pharamcological treatment of Alzheimer’s disease. Curr. Neuropharmacol..

[3] Pohanka M. (2011). Cholinesterases, A target of pharmacology and toxicology. Biomed. Papers.

[4] Pohanka M. (2012). Acetylcholinesterase inhibitors: A patent review (2008–present). Expert Opin. Ther. Pat..

[5] Martinez A., Castro A. (2006). Novel cholinesterase inhibitors as future effective drugs for the treatment of Alzheimer’s disease. Expert Opin. Inv. Drug.

[6] Schultes R.E. (1993). Plants in treating senile dementia in the Northwest Amazon. J. Ethnopharmacol..

[7] Mainen J.M., Donald F.O., Anke W. (2012). Ethnomedicine of the Kagera region, north western Tanzania. Part 3: Plants used in traditional medicine in Kikuku village, Muleba district. J. Ethnobio. Ethnomed..

[8] Fernando C., Luis O.R., Vanderlan D.S.B., Hosana M.D., Gabriela D.P. (2009). Piperamides and their derivatives as potential anti-trypanosomal agents. Med. Chem. Res..

[9] Da silva V.C., De Carvalho M.G. (2008). Chemical constituents from leaves of *Palicourea coriacea*. J. Nat. Med..

[10] Fadaei M., Hamid A.H.A. (2011). Antioxidant and antimicrobial activities of ferulic acid esters from *Ochrosia oppositifoli*. Malays. J. Sci..

[11] Zhang L.S.F., Yan L., Kong X.R. (2012). β-carboline alkaloids from the leaves of *Trigonostemon lii* Y.T. Chang. Bioorg. Med. Chem. Lett..

[12] Wong K.K., Ngo J.C., Liu S., Lin H.Q., Hu C., Shaw P.C., Wan D.C. (2010). Interaction study of two diterpenes, Cryptotanshinone and dihydrotanshinone, to human acetylcholinesterase and butyrylcholinesterase by molecular docking and kinetic analysis. Chem. Biol. Interact..

[13] Ahmed T., Gilani A.H. (2009). Inhibitory effect of curcuminoids on acetylcholinesterase activity and attenuation of scopolamine-induced amnesia may explain medicinal use of turmeric in Alzheimer’s disease. Pharmacol. Biochem..

[14] Obregon A.D.C., Schetinger M.R.C., Correa M.M., Morsch V.M., Da Silva J.E.P., Martins M.A.P., Bonacorso H.G., Zanatta N. (2005). Effects per se of organic solvents in the cerebral acetylcholinesterase of rats. Neurochem. Res..

