# Supplementary material for: Cholinesterase Enzymes Inhibitors from the Leaves of Rauvolfia Reflexa and Their Molecular Docking Study
Source: Molecules. 2013 Mar 25;18(4):3779–88. doi: 10.3390/molecules18043779 (PMC6270359; doi:10.3390/molecules18043779)

Supplementary Materials

Figure S1. ^{1}H-NMR spectrum of **1**.

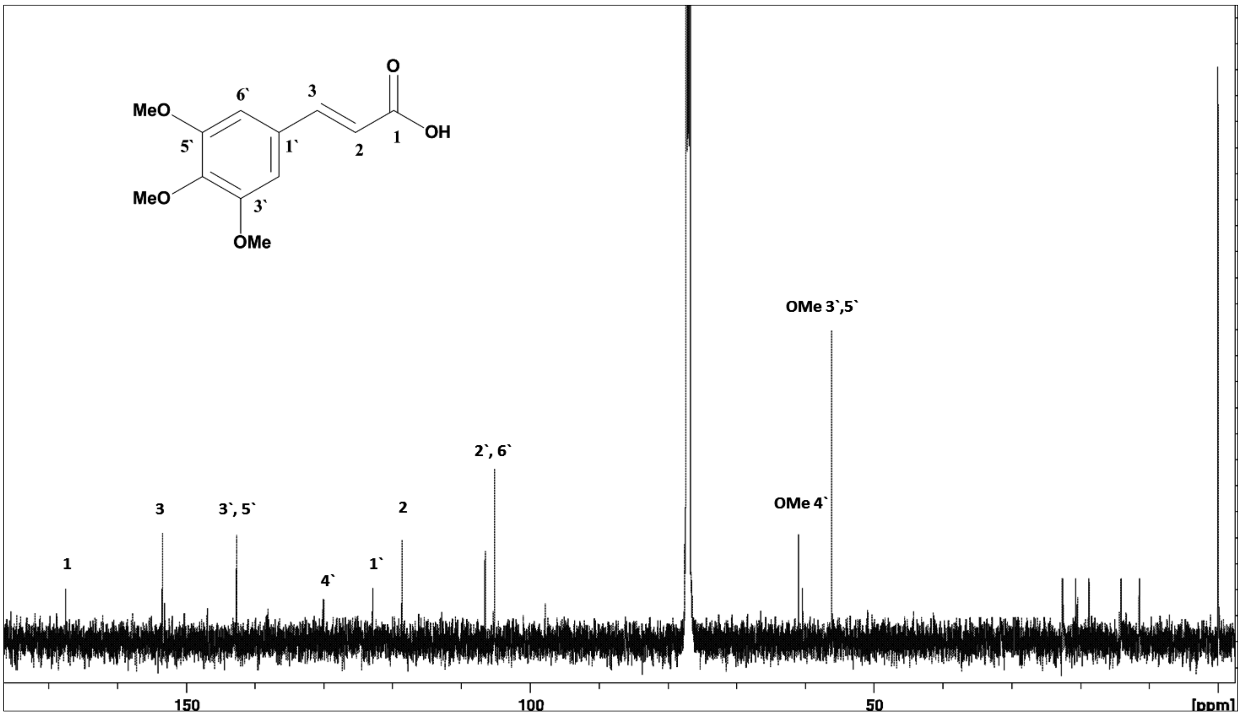

Figure S2. ^{13}C-NMR spectrum of **1**.

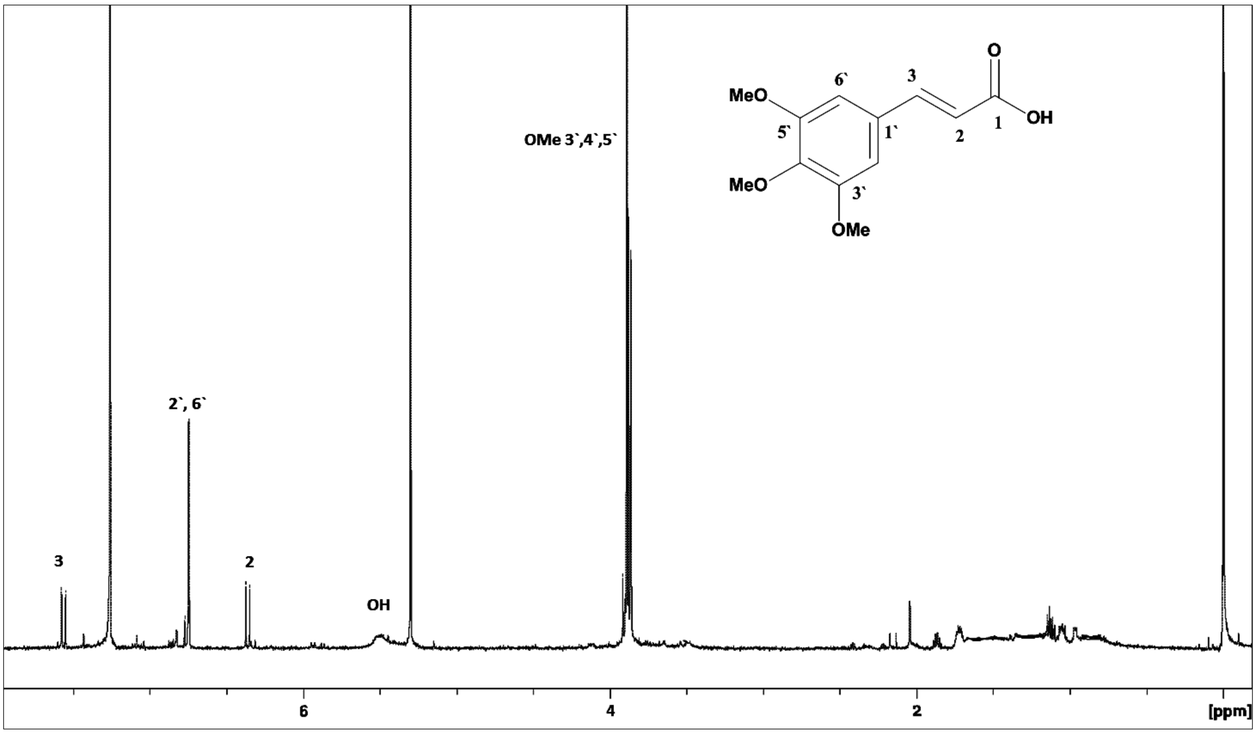

Figure S3. ^{1}H-NMR spectrum of **2**.

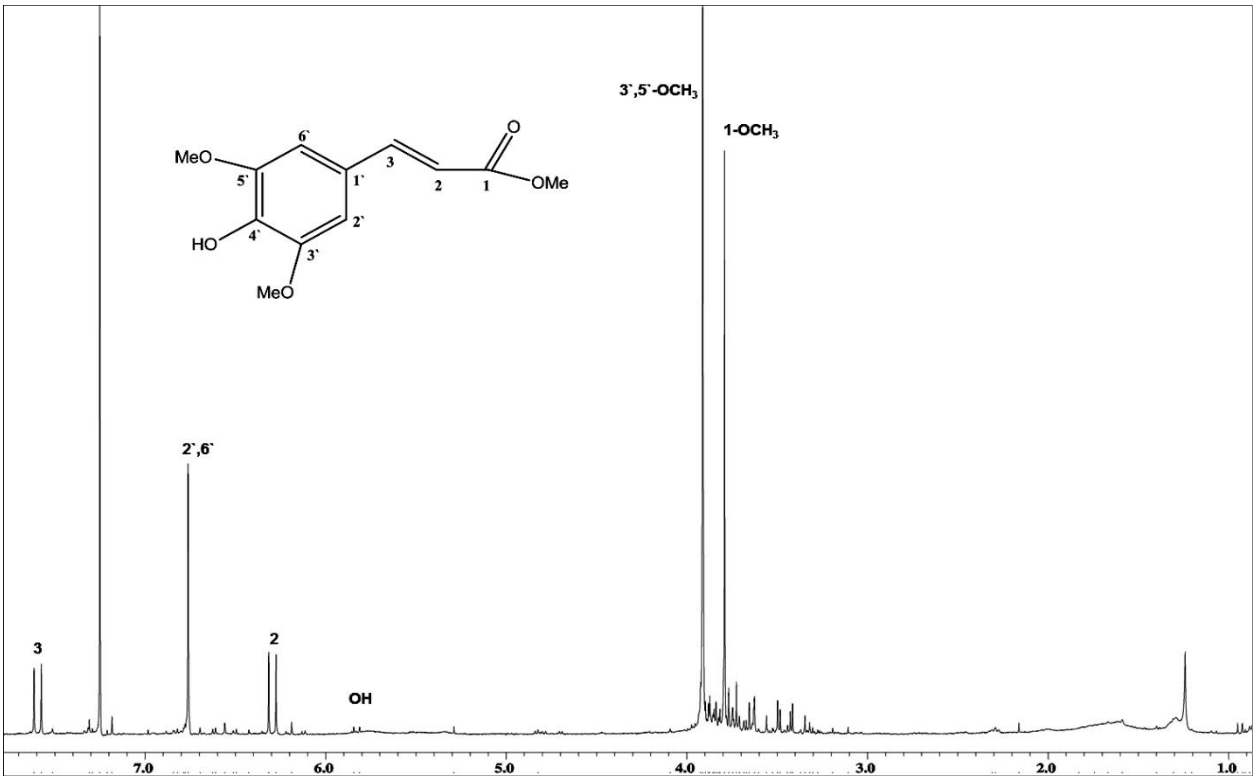

Figure S4. ^{13}C-NMR spectrum of **2**.

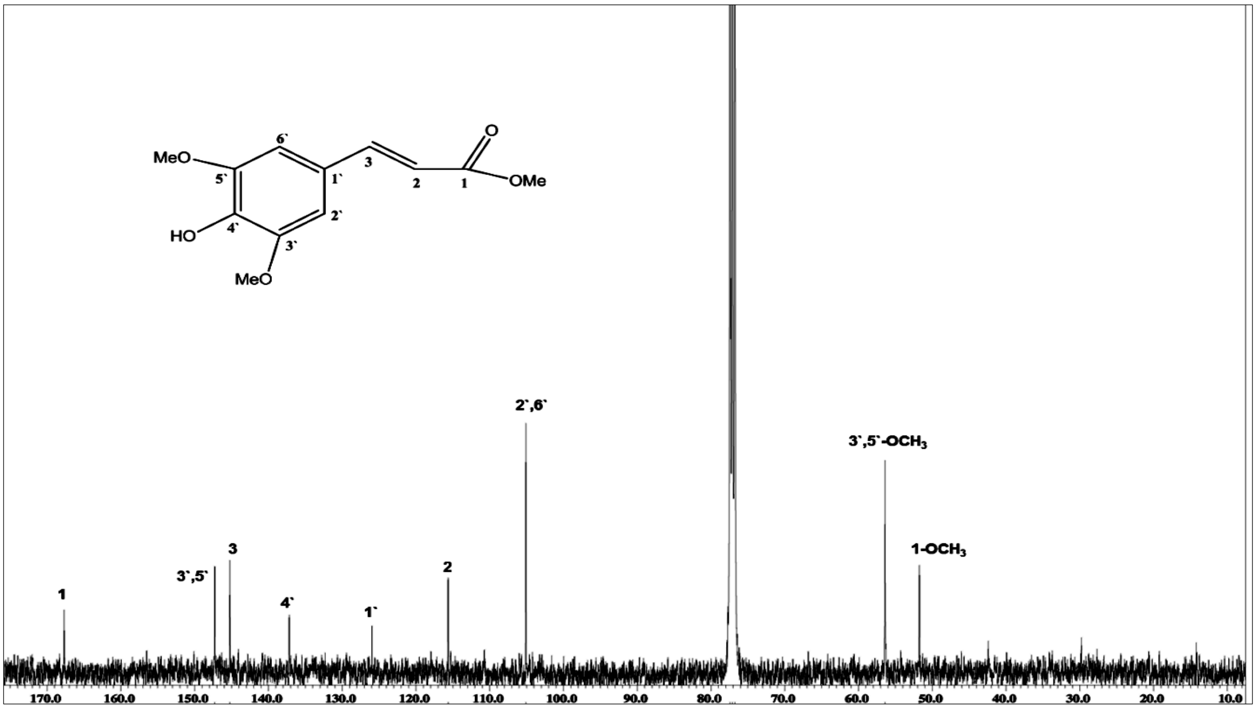

Figure S5. ^{1}H-NMR spectrum of **3**.

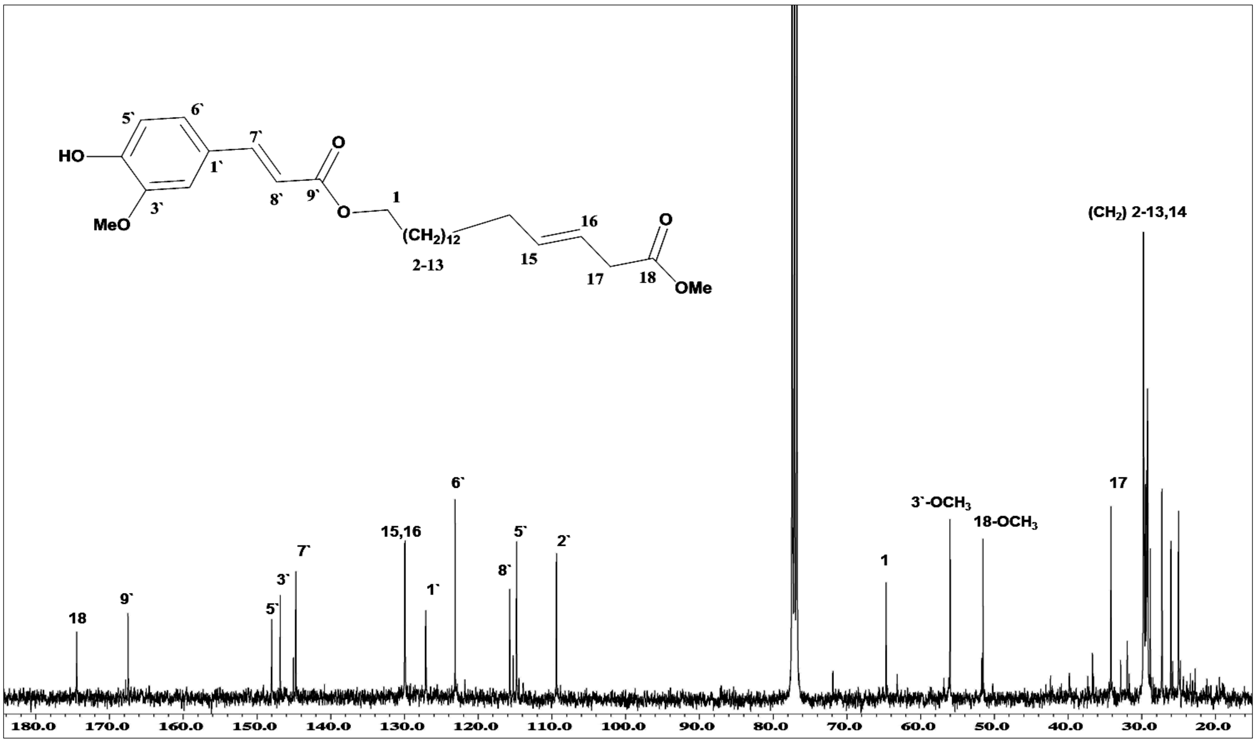

Figure S6. ^{13}C-NMR spectrum of **3**.

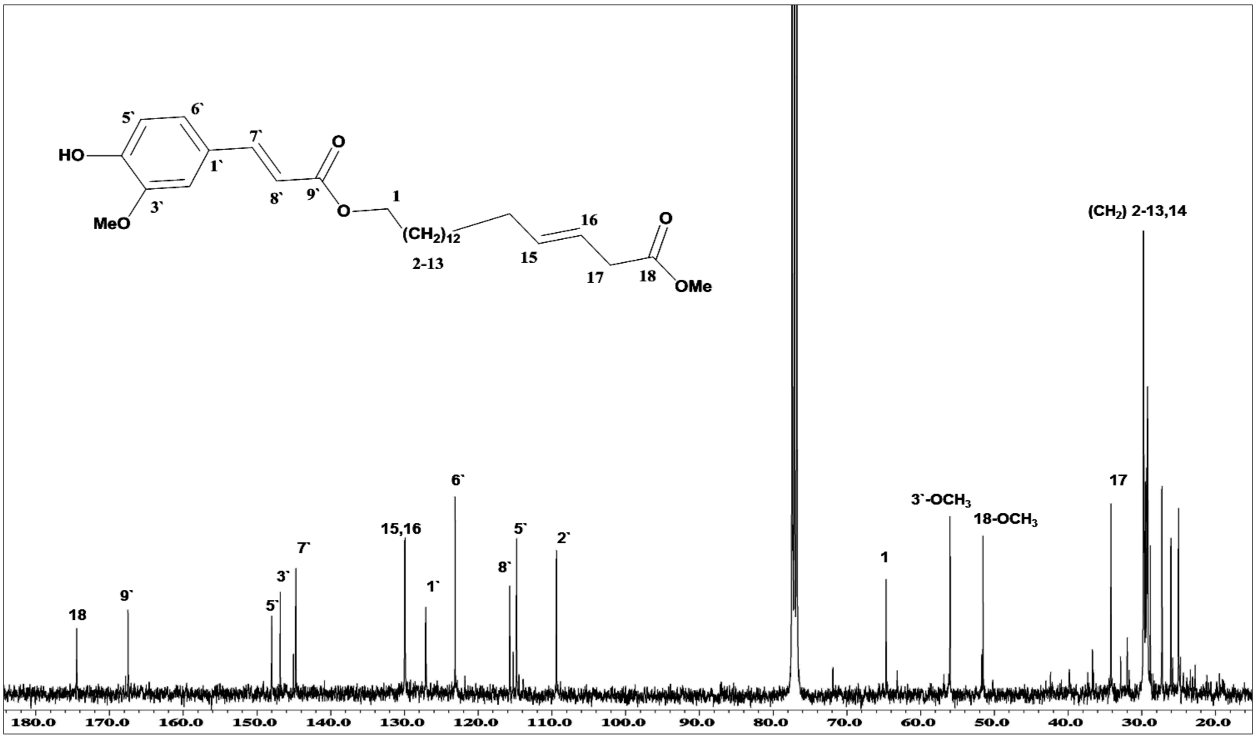

Figure S7. ^{1}H-NMR spectrum of **4**.

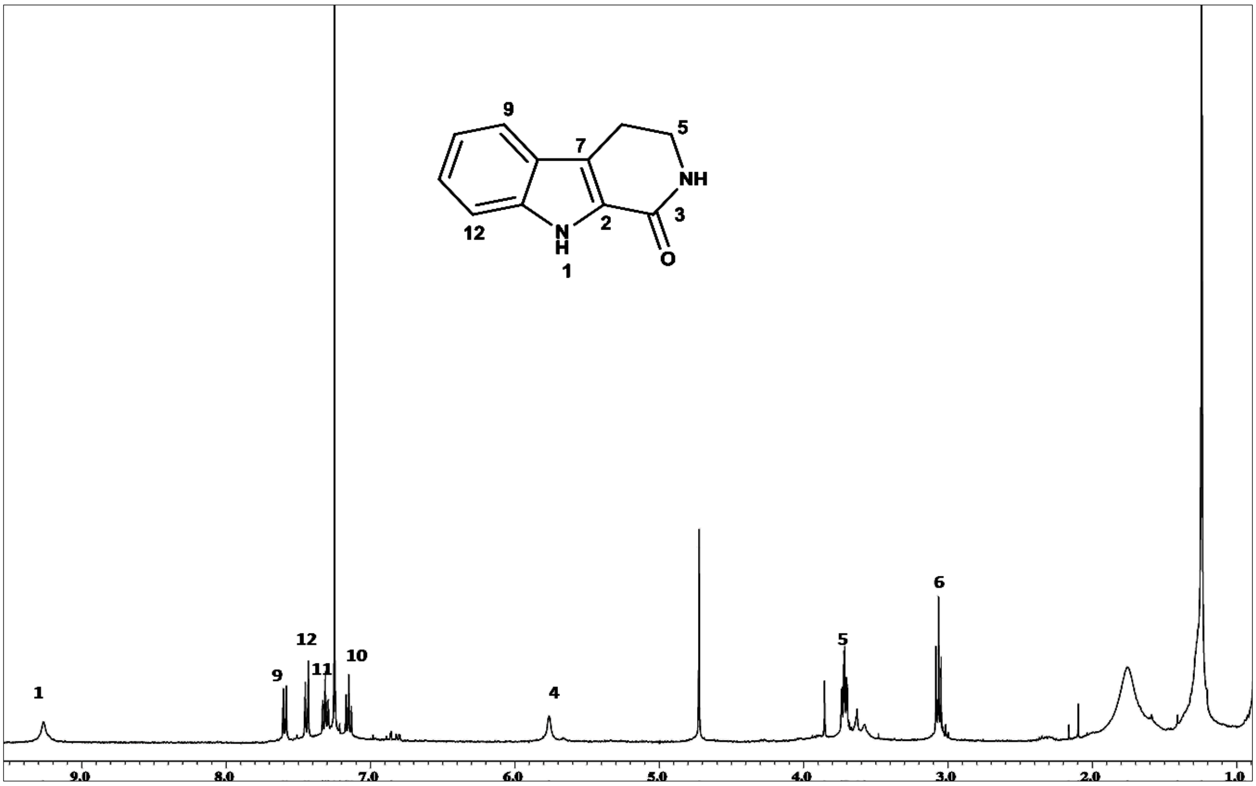

Figure S8. ^{13}C-NMR spectrum of **4**.

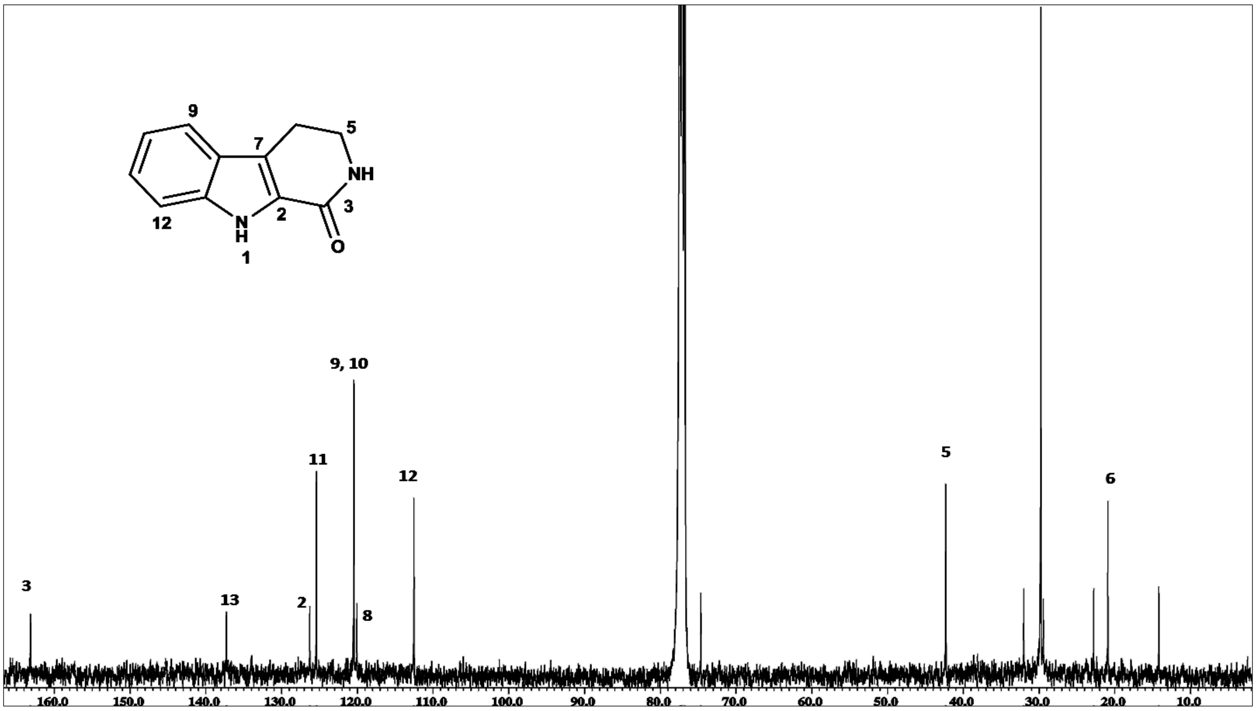

Supplement: Supplementary file 1 [file molecules-18-03779-s001.pdf]
